# Machine learning enhanced ultra-high vacuum system for predicting field emission performance in graphene reinforced aluminium based metal matrix composites

**DOI:** 10.1038/s41598-025-10946-7

**Published:** 2025-07-21

**Authors:** Sunil Kumar Pradhan, Subhayu Kabiraj, Shivin Kumar Gupta, Abhishek Singh, Padmakar G. Chavan, Shubham S. Patil, Trilok Nath Pandey

**Affiliations:** 1 School of Electronics Engineering, Vellore Institute of Technology, Chennai, 600127 India; 2 Department of Physics, School of Physical Sciences, Kavayitri Bahinabai Chaudhari North Maharashtra University, Jalgaon, 425001 India; 3 Department of Physics, Dr. Babasaheb Ambedkar Marathwada University, Aurangabad, 431004 MS India; 4 School of Computer Science Engineering, Vellore Institute of Technology, Chennai, 600127 India

**Keywords:** Field emission performance, Emission current stability, Graphene reinforced aluminum composites, Machine learning prediction, Ultra-high vacuum system, Engineering, Nanoscience and technology

## Abstract

The field emission performance of aluminium-based metal matrix composites reinforced with graphene (AlGr-MMCs) has garnered significant attention due to their potential applications in advanced electronics and in materials-based cathode systems. The field emission performance plays a crucial role in the high-power micro-wave tube devices and in energy applications, where material composition significantly influences emission stability and efficiency. This research work explores the impact of graphene incorporation into aluminum-based metal matrix composites (AlGr -MMCs) on field emission characteristics. By leveraging machine learning (ML) models, we predict the trends of emission current density (J) as a function of the applied electric field(E) and the emission current stability (I) over time(t) for Aluminium-Graphene (AlGr) composites with varying graphene weight% (wt%) greater than 1 and less than 2 (1.25, 1.5, 1.75, and 2.0). A two-stage machine learning framework was implemented. In Stage 1, datasets for pure aluminum, 0.5 wt% and 1.0 wt% graphene reinforced aluminium composites were used to train various ML models, categorized into five baskets: Decision tree-based, Support Vector models, Neural networks, Bayesian Models and Statistical Models. Model evaluation was conducted based on R²(R-squared), RMSE (Root Mean Squared Error), and Adjusted R² scores. In stage 2, the top models were further refined using advanced techniques, including Gradient-Based Methods and Ensemble Methods. Among the studied compositions, AlGr 2, containing 2 wt% graphene, exhibits the lowest turn-on electric field, whereas other compositions, including 1.25, 1.5, and 1.75 wt%, show comparatively higher values. This remarkable performance of AlGr2 arises from a delicate balance between conductive network formation, field enhancement and minimal agglomeration. The superior field emission performance of AlGr2 can be attributed to its optimal dispersion and percolation of graphene within the aluminium matrix. The findings demonstrate the efficacy of machine learning in accurately predicting field emission behavior, providing valuable insights for optimizing metal matrix composites in high-performance applications.

## Introduction

Metal matrix composites have been widely adopted in diverse technological fields, driven by their superior electrical, thermal and mechanical performance^1^. Their exceptional filed emission behavior and thermo-electrical properties render them indispensable for use in cathodes in high power microwave tube devices, nano and micro scale devices as well as in high-efficiency thermal energy harvesting systems^1–4^. Optimizing the performance of metal matrix composites necessitates careful analysis and fine-tuning of several critical parameters. Attributes such as the fraction of reinforcement, weight ratio, geometry and particle dimensions significantly impact the overall characteristics and behavior of the final composite material^5,6^. An essential aspect is the formation of bonds between the metal matrix and the dispersed phase, which can impart unique attributes to the composite structure and lead to remarkable alterations in its chemical and physical properties^7,8^. The distribution of the embedded phase within the metal matrix can be uniform or non-uniform and it can be controlled to achieve the desired results. Aluminum matrix composites (AMCs) are widely used across various industries due to their exceptional properties like high strength-to-weight ratio, high electrical and thermal conductivity, appreciable corrosion resistance and wear resistance. Aluminum matrix composites are currently being used in Aerospace Industry, Automotive and Electronics Industry and Biomedical Industry^9–12^. Aluminum offers a significant advantage over other commonly used metals due to its abundant availability, stemming from its widespread presence in the Earth’s crust. Additionally, aluminum possesses several unique properties, such as low density, superior thermal and electrical conductivity, high malleability, ductility, and excellent resistance to corrosion. However, the limitations of aluminum and its alloys include moderate mechanical strength and relatively low wear resistance^13^. Consequently, various reinforcement methods have been explored in which small amounts of aluminum oxide (Al_2_O_3_), boron nitride (BN), or silicon carbide (SiC) are integrated into aluminum, resulting in aluminum matrix composites with improved thermal, electrical, and mechanical characteristics^14–18^. Carbon nanotubes (CNTs) and graphene are increasingly utilized as reinforcement materials in aluminum composites due to their exceptional properties that significantly enhance the performance of the base material. Both CNTs and graphene offer remarkable mechanical strength and stiffness, with CNTs having a tensile strength far surpassing that of steel and graphene being the strongest material ever tested. This reinforcement leads to composites with superior tensile strength, hardness and overall structural integrity. Moreover, both CNT and graphene materials are extremely lightweight, complementing aluminum’s low density and making these composites ideal for applications in industries where weight reduction is essential, such as in aerospace^19–22^. The outstanding electrical and thermal conductivity of graphene and CNT improve the thermal management, electrical performance of the composites. This is particularly valuable for applications in electronics and power systems. The addition of CNTs and graphene also enhances wear resistance and reduces friction, increasing the durability of the composites^23–25^. When carbon nanotubes (CNTs) and graphene are used as reinforcements in aluminum matrix composites (AMCs), they both significantly enhance the mechanical, thermal, and electrical properties of the base aluminum. However, their effects vary due to their distinct structures and interaction mechanisms with the matrix. Graphene often outperforms CNTs in terms of load transfer efficiency due to its planar structure, offering better mechanical reinforcement when properly dispersed. However, CNTs may sometimes provide better dispersion in the matrix due to their 1D nature and aspect ratio, if properly functionalized^26–28^. Graphene generally provides superior thermal conductivity improvements due to better interfacial heat transfer and larger contact area with the matrix. That is why, Graphene usually performs better in electrical applications due to its large surface area and better percolation pathways in the composite. Graphene typically outperforms CNTs in wear and corrosion resistance due to better dispersion and barrier characteristics. Between graphene and CNT, graphene often achieves better reinforcement due to more efficient load transfer, but provided restacking is mitigated^29,30^. Both CNTs and graphene significantly enhance aluminum matrix composites, but graphene tends to provide better overall performance improvements, especially in mechanical strength, thermal/electrical conductivity, and wear resistance. However, the choice between them should be considered in terms of application-specific requirements, cost and availability and ease of processing and dispersion. If processing challenges are overcome, graphene-reinforced AMCs often outperform CNT-reinforced ones in most key metrics^41–43^.

This research work aims to predict the field emission performance of aluminium-based metal matrix composites reinforced with graphene (AlGr-MMCs) by analyzing trends in emission current density with respect to electric field (J-E) and current stability with respect to time (I-t) for varying graphene weight percentages (1.25, 1.5, 1.75, and 2.0). Using the existing experimental dataset for 1.0 wt% and 0.5 wt% of graphene inside Aluminium matrix and as well as pure Aluminum as a reference^1^, a two-stage ML framework was implemented. In stage 1, datasets for pure Aluminium, 0.5 wt% and 1.0 wt% graphene inside Aluminium matrix were used to train various models across five baskets: Decision Tree-based^32^, Support Vector Models, Neural Networks^33^, Bayesian Models^34^, and Statistical Models^35^. Gaussian Process Regressor (GPR) emerged as the best-performing model, followed by Support Vector Regressor (SVR)^36^, Random Forest^37^, and 3rd-degree Polynomial Regression. In Stage 2, advanced techniques such as Gradient-Based Methods (XGBoost, LightGBM, and CatBoost)^38–40^ and Ensemble Methods (Stacking, Voting, Bagging) were applied to refine predictions. The results demonstrate that ML provides an effective solution for modelling field emission in metal matrix composites, enabling accurate predictions for material optimization in high-performance applications^31^.

## Methodology

### Materials and preparation

The primary materials used in this study were aluminium powder (99.9% purity) and research grade graphene (2–5 layers, > 99.6% carbon), procured from Platonic Nanotech Private Limited. Aluminum powder is highly reactive with oxygen, leading to the formation of aluminium oxide (Al₂O₃), which can hinder its properties. To minimize this effect, the powder was handled and stored in an inert atmosphere (argon or nitrogen) using sealed containers and glove boxes. This approach was critical to preserving the reactivity of aluminium and preventing oxidation during preparation and processing. The preparation of the aluminium-graphene (AlGr) composites began with the dispersion of the powders. Aluminium and graphene were mixed in a zirconia jar containing zirconia balls in toluene. The balls to powder weight ratio was maintained at 20:1. In powder metallurgy, mixing aluminum and graphene in a zirconia jar with zirconia balls in toluene with a 20:1 ball-to-powder weight ratio is typically part of a ball milling process. Each component and condition serve a specific purpose. The ball milling is used to homogeneously disperse graphene within the aluminum matrix and improve the interfacial bonding between graphene and aluminum. It also achieves particle size reduction and refines the microstructure for better sinter ability. On the other hand, Zirconia (ZrO_₂_) is very hard and wear-resistant, minimizing contamination from the milling media. It is also chemically inert, especially important when working with reactive materials like aluminum and graphene. Zirconia is also considered to be non-magnetic, avoiding any unwanted reactions or phase changes caused by magnetic fields. Toluene in other hand, is a non-polar organic solvent, which is used as a wet milling medium for several reasons. It reduces oxidation of aluminum during milling by providing an inert liquid environment. It prevents cold welding of aluminum particles (a common issue due to Al’s ductility), it also Improves dispersion of graphene by reducing van der Waals attraction that causes agglomeration and finally it acts as a lubricant, reducing heat and friction during milling. The reason for balls-to-powder weight ratio (20:1) in the experiment can be considered as this ratio controls the energy input into the powder during milling. A 20:1 ratio can be considered in high-energy milling, which helps effectively break up agglomerated graphene, increase mixing intensity and reduce milling time and promote mechanical alloying between Al and graphene without damaging graphene structure excessively. High-energy ball milling (HBM) was performed at a rotational speed of 300 rpm for 10 h to achieve a homogeneous dispersion of graphene in the aluminium matrix^1^. The milled powders were compacted into green pellets using a cylindrical die with a length of 1.2 cm and a diameter of 0.6 cm. A hydraulic press applied a load of 1.30 tons for three minutes to ensure proper compaction. Subsequently, the green pellets were vacuum-sintered at 640 °C for 1.5 h with a controlled heating rate of 4 °C/minute^1^. This process facilitated thermal coalescence and produced AlGr composites with desirable mechanical and thermal properties. To achieve uniform graphene dispersion at 2 wt%, graphene was first ultrasonicated in ethanol to exfoliate the sheets, followed by gradual mixing with the base material under continuous stirring. The mixture was then dried carefully to prevent agglomeration. This controlled process ensured the formation of a stable, interconnected conductive network essential for efficient electron transport.

Schematic1 illustrates Aluminium-Graphene (AlGr) composites with varying graphene.

weight% (wt%) greater than 1 and less than 2 (1.25%, 1.5%, 1.75%, and 2.0%).

**Schematic 1:**
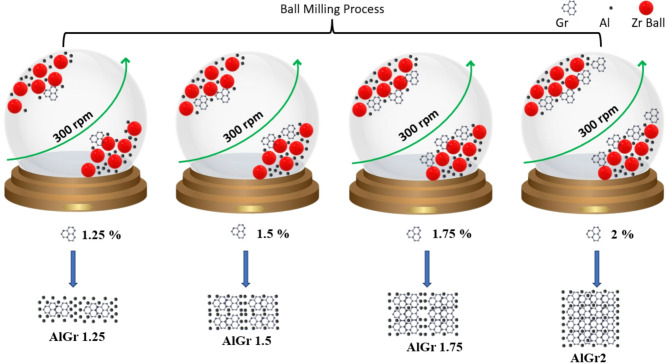
Synthesis method of AlGr composites at various wt% of Graphene greater than 1 and less than 2 (1.25%, 1.5%, 1.75%, and 2.0%).

### Computational modeling

In this study, a two-stage machine learning (ML) framework was developed to model and predict the field emission performance of graphene-reinforced aluminum-based metal matrix composites (AlGr- MMCs). The primary motivation behind this computational approach lies in the absence of any practical or experimental techniques capable of accurately predicting emission behavior for graphene concentrations exceeding 1 wt%. Machine learning, with its ability to recognize complex patterns and establish nonlinear relationships, provides a robust alternative for modeling field emission characteristics based on existing experimental datasets.

The modeling process began with the collection of experimental data for pure aluminum and aluminum composites reinforced with graphene at weight percentages of 0.5 wt% and 1.0 wt% ^1^. These datasets contained information on emission current density (J) as a function of applied electric field (E), as well as the stability of emission current(I) with respect to time(t). To create a structured dataset suitable for ML training, these two independent datasets were merged into a single format with four key parameters: emission current density (J), applied electric field (E), emission current stability (I), and time(t). This structured representation allowed the development of predictive models capable of estimating emission characteristics for higher graphene weight percentages, which were not included in the experimental dataset. Two distinct predictive scenarios were formulated to train the models effectively. In the first scenario, emission current density (J), applied electric field (E), and time(t) were treated as independent variables, while emission stability (I) was considered the dependent variable. This setup enabled the prediction of current stability under varying electric field conditions. In the second scenario, applied electric field (E), time(t) and emission current stability (I) were used as independent variables, while emission current density (J) was considered the dependent variable, allowing for the estimation of current density at different field strengths and emission stabilities.

To ensure a comprehensive evaluation of predictive performance, multiple machine learning models were implemented and categorized into five major groups based on their underlying principles. The Decision Tree-Based Models, including Random Forest, Extra Trees, and Decision Tree were applied to capture hierarchical relationships in the dataset. The Support Vector Models, specifically Support Vector Regression (SVR) and Nu-SVR, were utilized to handle nonlinear dependencies between field emission parameters. Neural Network models, such as Multi-Layer Perceptron (MLP) and Feedforward Neural Network (FNN) were explored to leverage deep learning techniques for recognizing complex emission trends. Bayesian Models, including Gaussian Process Regressor (GPR) and Bayesian Ridge were introduced to incorporate probabilistic approaches for estimating emission behavior. Additionally, Statistical Models, such as Monte Carlo Simulation, Polynomial Regression, and Holt-Winters Method, were employed to establish mathematical relationships and extrapolate field emission properties.

The initial selection of machine learning models for Stage 1 was guided by both domain knowledge and preliminary benchmarking. Five categories like Decision Tree-based models, Support Vector Machines (SVMs), Neural Networks, Bayesian Models, and Statistical Methods were chosen to represent a broad spectrum of regression strategies. These categories were selected based on their proven performance in handling small-to-medium scientific datasets, nonlinear dependencies, and experimental noise characteristics inherent to field emission data. Within each category, multiple algorithms were tested. The final model selected from each group like Random Forest (Decision Trees), SVR (Support Vector Machines), GPR (Bayesian), MLP/FNN (Neural Networks), and Polynomial Regression (Statistical) was chosen based on its superior performance on internal validation metrics (MSE, RMSE, R²). This two-stage selection process (by basket, then refinement in Stage 2) ensured a balanced comparison across algorithmic families, enabling downstream ensemble learning while maintaining interpretability. Simpler baselines (e.g., linear regression) were initially evaluated but excluded due to poor performance in capturing nonlinearities observed in the JE and Stability datasets. Thus, the selection process combined practical performance with theoretical justification from existing literature and emission modeling challenges.

To ensure optimal model performance and prevent overfitting, each machine learning model underwent hyperparameter tuning based on literature-guided defaults and performance trials on the validation set. In Stage 1, baseline hyperparameters were used for comparative evaluation across five model categories. The Random Forest Regressor was initialized with n_estimators = 100 and max_depth = None, while Extra Trees and Decision Tree models used similar default parameters to benchmark tree-based performance. Support Vector Regression (SVR) was configured with a radial basis function (RBF) kernel, and hyperparameters C = 1.0 and epsilon = 0.1, tuned via grid search in initial trials. Nu-SVR followed similar settings. For neural models, the Multi-Layer Perceptron (MLP) was implemented with hidden_layer_sizes=(64, 64), activation=’logistic’, and max_iter = 500. A custom Feedforward Neural Network (FNN) was also designed using TensorFlow Keras, consisting of two hidden layers with 64 neurons each and sigmoid activations, trained using the Adam optimizer and Mean Squared Error loss function for 100 epochs. Bayesian Ridge Regression and Gaussian Process Regression (GPR) were used with default configurations due to their internal regularization, with GPR employing a RBF kernel. Statistical models included third-degree Polynomial Regression with Bayesian Ridge as the linear estimator, and Holt-Winters Exponential Smoothing configured with additive trend and no seasonality. Monte Carlo simulations were conducted with 1000 bootstrapped resamples using a Random Forest base estimator to assess probabilistic stability.

In Stage 2, more advanced hyperparameter tuning was employed. XGBoost, LightGBM, and CatBoost were configured with n_estimators = 500, learning_rate = 0.01, and max_depth = 7, with additional regularization via subsample = 0.95 and colsample_bytree = 0.95 for gradient-boosted methods. CatBoost used l2_leaf_reg = 3 for L2 regularization. Ensemble methods were constructed using: (a) Stacking: with XGBoost, LightGBM, and CatBoost as base models and Ridge Regression (alpha = 0.5) as the final estimator. (b) Voting: with a weight distribution of 0.3 (XGB), 0.3 (LGB), and 0.4 (Cat). (c) Bagging: employed 50 Decision Trees with a maximum depth of 7. All models were trained on normalized input data using either StandardScaler or MinMaxScaler to ensure convergence and comparability. Performance was evaluated via RMSE, R², and Adjusted R² metrics, with 5-fold and 10-fold cross-validation used to test generalizability.

Once the models were trained, their performance was evaluated using several statistical metrics to determine their predictive accuracy. Key metrics included Mean Squared Error (MSE), which quantified the average squared difference between predicted and actual values, Root Mean Squared Error (RMSE), which provided a measure of error magnitude, R-squared (R^2^, which indicated how well the independent variables explained the variance in the dependent variable and Adjusted R-squared, which accounted for model complexity and prevented over fitting. These metrics allowed for an objective comparison of different models to identify the most reliable predictive approach.

**Fig. 1:**
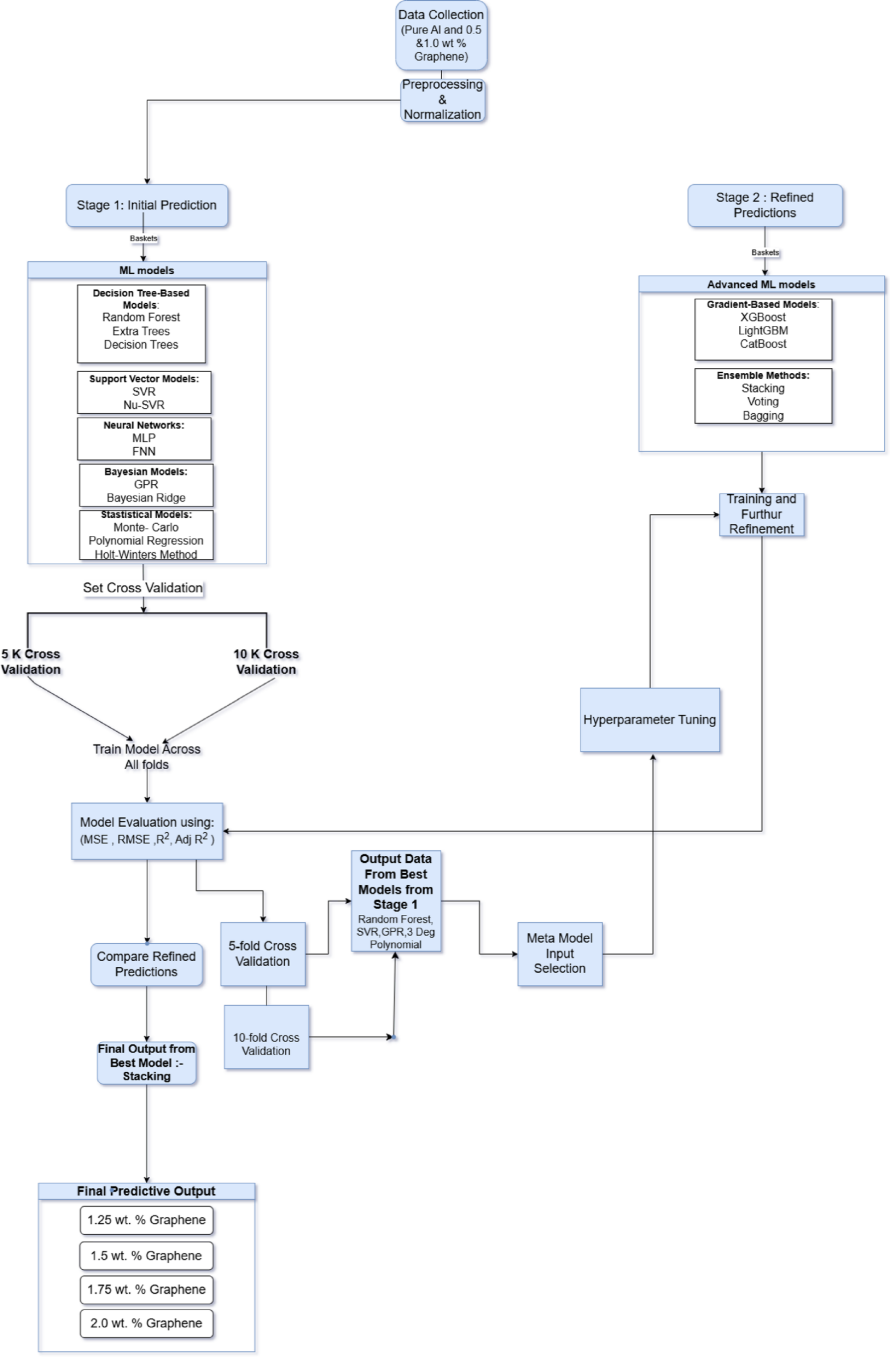
Stages of ML algorithm to determine the predictive analysis of AlGr composites.

The computational framework was designed as a two-stage process. In the stage 1, the models were trained using the experimental dataset consisting of pure Aluminum, 0.5 wt% of graphene inside Aluminium matrix and 1.0 wt% graphene inside Aluminium matrix and the evaluation was done based on their statistical performance. The best-performing model from each category was selected for further refinement in the stage 2, where more advanced computational techniques were applied. Gradient-based boosting methods, including XGBoost, LightGBM and CatBoost were introduced to improve the predictive efficiency of the selected models by refining decision trees and optimizing hyper parameters. Ensemble learning techniques, such as Stacking, Voting, and Bagging, were incorporated to enhance model robustness by combining multiple predictions and reducing over fitting. A flowchart is given in Fig. 1 to understand the stages of ML algorithm used. These advanced methodologies enabled the development of a predictive framework capable of extending learned trends in field emission behavior to higher graphene weight percentages (1.25–2.0 wt%) in a physically consistent manner, based on smooth extrapolations from lower experimentally validated regimes.

### Data analysis and validation

To establish the credibility of the machine learning predictions, rigorous data analysis and validation techniques were applied. The accuracy of predictive models is highly dependent on the quality and consistency of the input data. So, Preprocessing steps were undertaken to ensure data integrity. The experimental dataset for each composite concentration was derived from two primary sources: the current density vs. electric field data and the current Stability data. The current density vs. electric field data contained 65 data points per concentration and included the following features: applied voltage (V), current (I), ten-times current (I×10), current density (J), electric field (E), inverse electric field (1/E), squared electric field (E²), and the natural logarithm of J/E² (ln(J/E²)). The Current Stability data comprised 1800 data points per concentration, tracking emission current (in microamperes) over time (in minutes). From these two datasets, four critical features like electric field (E), current density (J), emission current (I), and time (t) were extracted and compiled into a unified training dataset for each material composition: Pure Aluminum, AlGr0.5, and AlGr1. These unified datasets served as the foundation for both Stage 1 and Stage 2 of machine learning model training. It is important to note that the raw experimental data used to generate these structured datasets were sourced directly from our previous work^1^. While the paper does not include the full dataset in tabular form, the corresponding author of the present manuscript is also the corresponding author of the cited paper and retains access to the complete experimental measurements. These raw data were carefully processed—by extracting electric field, current density, emission current, and time—to construct machine learning-compatible datasets used in this study. Prior to training, all data were subjected to normalization using the Min-Max scaling method to bring the feature values within a uniform range and reduce training bias. Outliers, particularly in emission current data, were identified using the Interquartile Range (IQR) method and removed to enhance data quality. This preprocessing ensured consistency, comparability across concentrations, and robustness of the model predictions.

A robust validation strategy was implemented to evaluate the generalization capabilities of the predictive models. The dataset was systematically divided into training and testing subsets, with the training data used to develop the models and the testing data used to assess their predictive accuracy. A cross-validation approach was employed, in which the dataset was repeatedly split into different training and validation sets to ensure that the models did not memorize specific patterns but instead learned generalizable relationships. This strategy minimized the risk of overfitting and allowed for a more reliable estimation of model performance. To quantify the accuracy of predictions, multiple statistical validation metrics were employed. Root Mean Squared Error (RMSE) was used to measure the average deviation between predicted and actual values, providing an indication of the error magnitude. The R-squared (R^2^ coefficient was calculated to determine how well the independent variables explained the variance in the dependent variable, with higher values indicating better predictive accuracy. Additionally, Adjusted R-squared was incorporated to account for the number of predictors in the model and to ensure that added complexity contributed to genuine improvements in prediction quality rather than overfitting. These metrics provided an objective assessment of model performance and allowed for the identification of the most reliable predictive approaches.

To rigorously evaluate the generalization capability of the predictive models, both 5-fold and 10-fold cross-validation strategies were employed during training and performance assessment. The datasets were randomly shuffled prior to each split to prevent order-based bias, and a fixed random_state = 42 was used to ensure reproducibility of results across runs. Given the regression nature of the problem and the continuous distribution of field emission variables, stratified splitting was not applied. Instead, each fold preserved the global distribution of target variables (emission current and current density) by random allocation. For every fold, the training subset was used to fit the model while the held-out fold served as the validation set. This process was repeated iteratively across all folds, ensuring that every data point was included in a validation set exactly once. The average performance across folds was then computed to obtain a robust estimate of the model’s predictive power and stability on unseen data.

To further validate the robustness of the machine learning models, predictive stability tests were conducted across different graphene concentrations. The trained models were applied to aluminum composites with graphene weight percentages of 1.25 wt%, 1.5 wt%, 1.75 wt%, and 2.0 wt%, the values that were beyond the range of the experimental dataset. By comparing the predicted field emission characteristics at these higher graphene concentrations with expected trends, the stability and reliability of the machine learning framework were assessed. This step was crucial in confirming that the developed models could successfully generalize beyond the training data and provide meaningful insights for material optimization in practical applications.

Through this comprehensive methodology, a reliable and scalable computational framework was established for predicting the field emission behavior of graphene-reinforced aluminum composites. The two-stage machine learning approach enabled accurate modeling of emission trends beyond 1 wt% of graphene, a region where experimental data is currently unavailable. By leveraging advanced ML techniques, this study provides a powerful tool for researchers and engineers to optimize field emission performance in advanced materials, paving the way for future applications in high-performance electronics and energy systems.

## Results and discussion

The performance of all models was quantified using four primary metrics: Mean Squared Error (MSE), Root Mean Squared Error (RMSE), R², and Adjusted R². To assess consistency, each model’s evaluation was repeated across both 5-fold and 10-fold cross-validation regimes. This dual-layered approach enabled comparative analysis of not only average performance but also variability across folds. The best-performing models, including Gaussian Process Regressor (GPR), Support Vector Regression (SVR), and ensemble methods such as Stacking, consistently yielded high R² scores (above 0.93) with low standard deviations (σ < 0.5 in RMSE across folds), indicating excellent stability and robustness. This confirms that the predictions were not overly dependent on any particular subset of the data. The superiority of ensemble methods in Stage 2 was particularly evident, with the Stacking model demonstrating the most balanced performance across all metrics, regardless of fold count. Supplementary results include fold-wise RMSE and R² distributions for key models, providing further statistical evidence of reliability. These insights validate that the proposed ML framework can generalize well to field emission data beyond the training range, particularly for extrapolations to higher graphene content. Table 1 contains the Baskets of algorithms used in Stage 1.

**Table 1:** Different ML algorithm in stage 1.

S. No.	Models used	Algorithms
1	Decision Tree-Based Models	(Random Forest, Extra trees, Decision Tree)
2	Support Vector Models	(SVR, Nu-SVR)
3	Neural Networks	(MLP, FNN)
4	Bayesian Models	(Gaussian Process Regressor (GPR), Bayesian Ridge)
5	Statistical Models	(Monte-Carlo, Polynomial Regression, Holt-Winters Method)

The study systematically categorized machine learning models into five broad groups based on their computational techniques and predictive approaches, selecting the best-performing model from each category. In this consideration, the Decision Tree-Based Models, Random Forest emerged as the most effective and leveraging its ensemble learning mechanism to aggregate multiple decision trees for more accurate and stable predictions. It achieved a Mean Squared Error (MSE) of 265.17 and an R² score of 0.8528, indicating a strong ability to model complex relationships in the dataset while minimizing over fitting. Extra Trees and Decision Tree, the other models in this category, showed inferior performance, with significantly higher error values, reinforcing the robustness of Random Forest in handling nonlinear dependencies in the data.

In the Support Vector Models category, SVR (Support Vector Regression) stood out as the best-performing model, surpassing Nu-SVR in accuracy. SVR effectively mapped input features to higher-dimensional spaces using kernel functions, allowing it to capture nonlinear patterns more efficiently than simple regression methods. It recorded an MSE of 235.80 and an R² score of 0.8614, making it the second most accurate model in the study. This performance was superior to Nu-SVR, which exhibited slightly higher error values and a lower ability to generalize across the dataset.

The Neural Networks category, consisting of Multi-Layer Perceptron (MLP) and Feedforward Neural Network (FNN), did not produce strong candidates. Both models exhibited poor performance due to their high computational complexity and potential over fitting issues. MLP recorded an MSE of 1115.40 with an R² score of 0.5633, while FNN showed an MSE of 695.91 with an R² score of 0.7053. These results indicate that the neural network models struggled to generalize well for this dataset, possibly due to the limited amount of training data or improper hyper parameter tuning, making them unsuitable for this application.

Among the Bayesian Models, the Gaussian Process Regressor (GPR) demonstrated the highest accuracy, making it the most reliable model in this study. The GPR uses probabilistic modeling to estimate functions that best fit the data, incorporating uncertainty quantification for robust predictions. It significantly outperformed Bayesian Ridge Regression, achieving the lowest MSE of 215.80 and the highest R² score of 0.8876. This outstanding performance indicates that GPR not only captured the nonlinear dependencies effectively but also generalized well across different graphene concentrations, making it the best model overall.

In the statistical model category, 3rd degree Polynomial Regression (Poly3) performed the best, showcasing an ability to fit nonlinear trends in the data. It recorded an MSE of 286.75 and an R² score of 0.8455, positioning itself as a reasonably strong model. However, compared to advanced machine learning approaches like GPR and SVR, it lacked the flexibility to handle more complex variations in emission behavior. Other statistical models, such as Monte Carlo Simulation and Holt-Winters Method, performed significantly worse, with high error values and poor predictive power.

When comparing the best models from each category, Gaussian Process Regressor (GPR) emerged as the most effective and reliable model, outperforming all others in terms of predictive accuracy. Its ability to model complex nonlinear relationships while incorporating uncertainty estimation made it particularly well-suited for predicting field emission performance in metal matrix composites. Following GPR, SVR ranked as the second-best model, demonstrating strong predictive capability through its use of kernel functions, which allowed it to manage high-dimensional data efficiently. Random Forest secured the third position, benefiting from its ensemble learning strategy, which helped mitigate over fitting while ensuring stable performance. However, it exhibited slightly higher error values compared to GPR and SVR. The 3rd-degree Polynomial Regression took the fourth spot, proving useful for modeling some nonlinear dependencies but lacking the adaptability and precision of the top three learning models. Table 2 presents a detailed and a bucket wise overview of the various parameters associated with the Algorithms utilized in Stage 1.

**Table 2:** Depiction of different parameters of ML algorithms in stage 1. Since RMSE measures the average magnitude of prediction errors, lower values indicate better model performance. Among all the models, Gaussian process regressor (GPR) demonstrates the lowest RMSE, making it the most effective model in terms of prediction accuracy. Following closely behind are support vector regression (SVR) and random forest, which also show relatively low RMSE values, indicating their strong predictive capabilities. The R square and adjusted R squared value of GPR is higher than other models. Table 3 contains the baskets of algorithms used in stage 2.

Base Model	Basket number	Basket name	MSE	RMSE	R2	Adj R2
GPR	4	Bayesian Models	215.8046329	14.69029043	0.887622791	0.887373341
SVR	2	Support Vector Models	235.8049834	15.35594293	0.861418319	0.861110702
RandomForest	1	Decision Tree-Based Models	265.1747244	16.28418633	0.852849125	0.852522485
Poly3	5	Statistical Models	286.759422	16.93397242	0.845503831	0.845160887
BayesianRidge	4	Bayesian Models	299.0621907	17.29341466	0.82660697	0.82622208
NuSVR	2	Support Vector Models	301.1729789	17.35433602	0.811156993	0.810737807
ExtraTrees	1	Decision Tree-Based Models	326.5911118	18.071832	0.790315019	0.78984957
Monte_Carlo	5	Statistical Models	527.6887581	22.97147706	0.728778059	0.728176012
FNN	3	Neural Networks	695.9155292	26.38021094	0.705334504	0.704680419
DecisionTree	1	Decision Tree-Based Models	1152.492558	33.94838079	0.602546309	0.601664059
MLP	3	Neural Networks	1115.405659	33.39768942	0.563393164	0.562424003
HoltWinters	5	Statistical Models	1179.041508	34.33717384	−0.000197328	−0.002417521

**Table 3:** Models used in Stage-2 for further refining the prediction.

S No.	Model used	Algorithm
1	Gradient Based	(XGBoost, LightGBM)
2	Ensemble Method	(Stacking, Voting, Bagging)

In Stage 2, with cross validation of 5k, the best-performing models from Stage 1, Gaussian Process Regressor (GPR), Support Vector Regressor (SVR), Random Forest, and 3rd-degree Polynomial Regression were further refined using advanced gradient-based methods and ensemble learning techniques to enhance predictive accuracy. The primary objective was to leverage these advanced machine learning methodologies to improve generalization, minimize errors, and optimize predictive stability for modelling field emission performance in graphene-reinforced aluminium-based metal matrix composites (AlGr-MMCs). To ensure a robust and unbiased evaluation, the models were subjected to cross validation of 5k, allowing for a comprehensive assessment of their generalizability. The key performance metrics used for comparison were Mean Squared Error (MSE), Root Mean Squared Error (RMSE), R-squared (R²), and Adjusted R².

In this stage, two categories of machine learning techniques were employed. The Gradient-based methods, including XGBoost, LightGBM, and CatBoost, were applied to improve feature learning through boosted decision trees. Alongside these, ensemble learning approaches, such as Stacking, Voting, and Bagging, were used to aggregate multiple models and enhance predictive power. The results from this refinement stage revealed that Stacking outperformed all other models, achieving the lowest MSE (155.846) and RMSE (12.48383), while maintaining the highest R² (0.933962) and Adjusted R² (0.93160372). This clearly indicates that Stacking effectively leveraged the combined strengths of multiple models, leading to the best predictive performance.

Among the gradient-based models, CatBoost-GPR emerged as the strongest, achieving an MSE of 167.509, closely followed by XGBoost-GPR (MSE: 174.8936) and LightGBM-GPR (MSE: 180.1992). The integration of Gaussian Process Regression (GPR) with boosting techniques proved highly effective, as these models demonstrated significantly lower error rates compared to their counterparts. The Support Vector Regression (SVR)-based models, such as CatBoost-SVR and XGBoost-SVR, also performed well but did not reach the same level of accuracy as the GPR-based models.

In contrast, ensemble methods like Voting and Bagging provided moderate improvements but did not surpass the performance of Stacking. The Voting-based model recorded an MSE of 197.5356, striking a balance between bias and variance, whereas Bagging models, including Bagging-RF and Bagging-SVR, showed higher MSE values, making them relatively less favourable. Notably, polynomial regression models, which had shown promise in Stage 1, underperformed in Stage 2, with Bagging-Polynomial yielding the highest MSE (263.919). This suggests that polynomial models struggle to capture the complex nonlinear dependencies required for accurate field emission predictions, making them suboptimal for this study.

The findings from Stage 2 confirm that Stacking is the optimal model for predicting field emission performance in AlGr-MMCs by giving approximate 93.39% accuracy result. By combining multiple models in a hierarchical manner, stacking significantly reduced prediction errors and improved generalization, making it the most reliable approach. The results further validate the effectiveness of advanced ensemble techniques over standalone models and traditional boosting approaches. This research establishes a robust computational framework for predicting field emission performance, facilitating precise material optimization for high-performance electronics and energy applications. The successful implementation of Stacking highlights the potential of machine learning in advancing materials science, demonstrating its ability to uncover complex nonlinear relationships that are otherwise difficult to model through conventional experimental techniques. Table 4 shows the different measured matrices of stage 2 with a cross validation of 5k.

**Table 4:** An overview of the various evaluation metrics measured in stage 2, incorporating a 5-fold cross-validation process.

Model	MSE	RMSE	*R* ^2^	Adjusted *R*^2^
Stacking	155.846	12.48383	0.933962	0.9316037
CatBoost_GPR	167.509	12.94253	0.921666	0.9188685
XGBoost_GPR	174.8936	13.22473	0.915848	0.9128422
CatBoost_SVR	182.6717	13.51561	0.902046	0.8985476
LightGBM_GPR	180.1992	13.42383	0.901962	0.8984608
XGBoost_SVR	190.8437	13.81462	0.891065	0.8871748
CatBoost_Polynomial	224.5423	14.98473	0.885848	0.8817708
LightGBM_SVR	195.4927	13.98187	0.88414	0.8800025
CatBoost_RF	205.1348	14.32253	0.881666	0.8774399
XGBoost_RF	214.2011	14.63561	0.881046	0.8767976
Voting	197.5356	14.05473	0.875848	0.8714137
XGBoost_Polynomial	230.8764	15.19462	0.871065	0.8664605
LightGBM_RF	219.4496	14.81383	0.870962	0.8663537
Bagging_RF	245.2942	15.66187	0.85414	0.8489311
LightGBM_Polynomial	236.6222	15.38253	0.851666	0.8463685
Bagging_SVR	219.2061	14.80561	0.832046	0.8260476
Bagging_GPR	201.5154	14.19561	0.831846	0.8258405
Bagging_Polynomial	263.9199	16.24561	0.822046	0.8156905

**Fig. 2:**
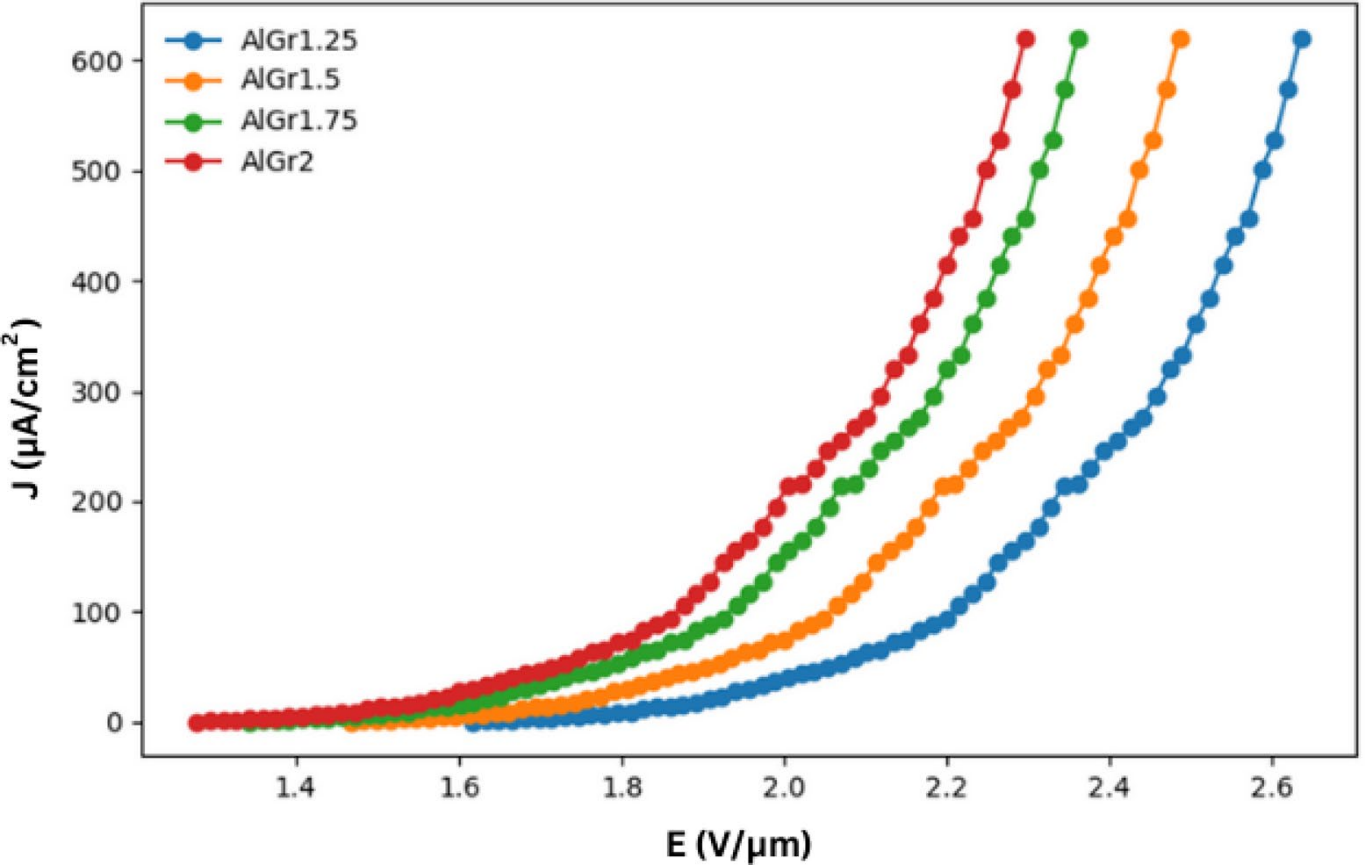
Field emission current density (J) with respect to applied field (E) at various wt% of Graphene greater than 1 and less than 2 (1.25%, 1.5%, 1.75%, and 2.0%) inside Aluminium matrix.

**Fig. 3:**
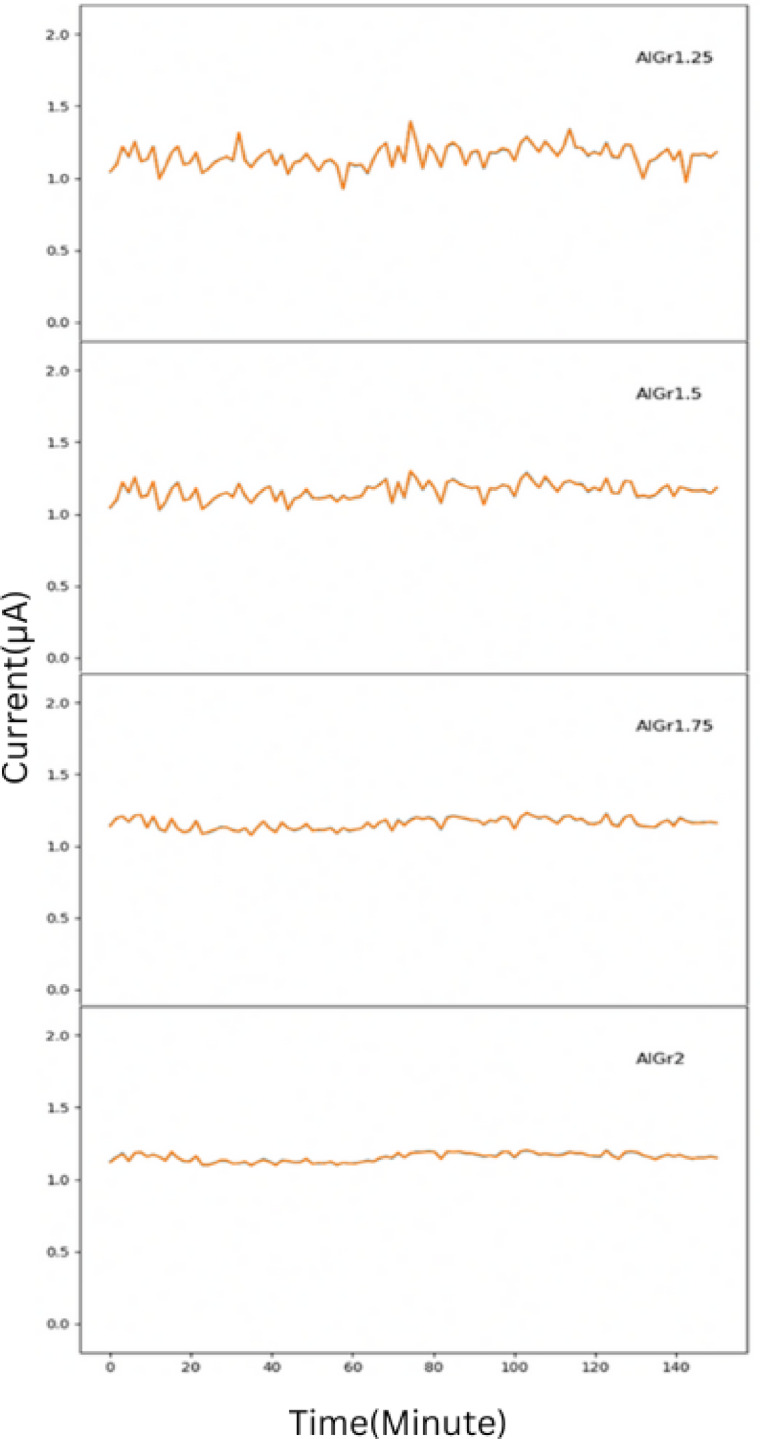
Plot for emission current stability(I) with respect to time(t) using stacking for the samples AlGr1.25, AlGr1.5, AlGr1.75, and AlGr2.

**Fig. 4:**
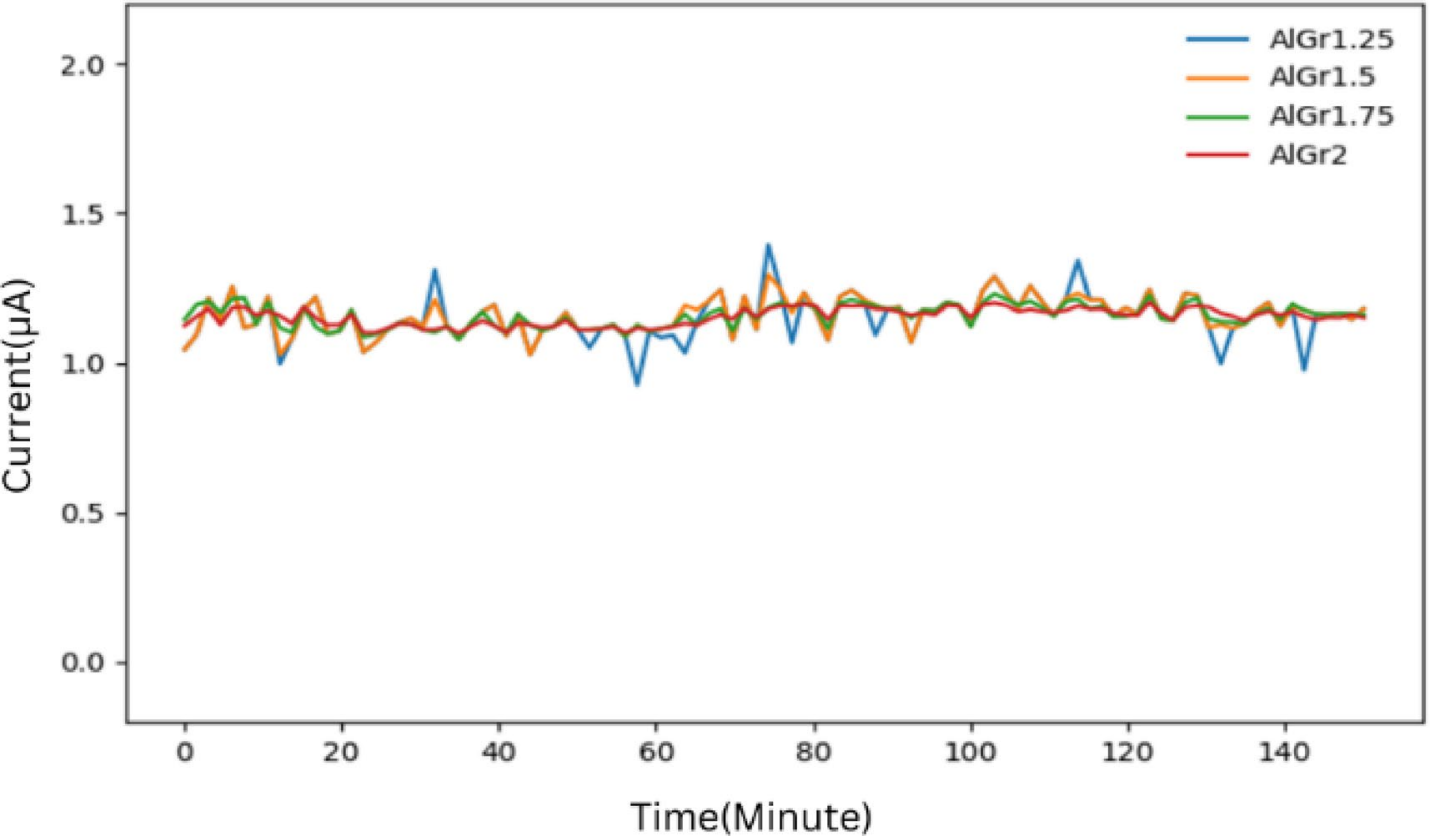
Emission current (I) with respect to time (t) for AlGr1.25, AlGr1.5, AlGr1.75 and AlGr2 recorded for the preset value of 1 mA.

**Fig. 5:**
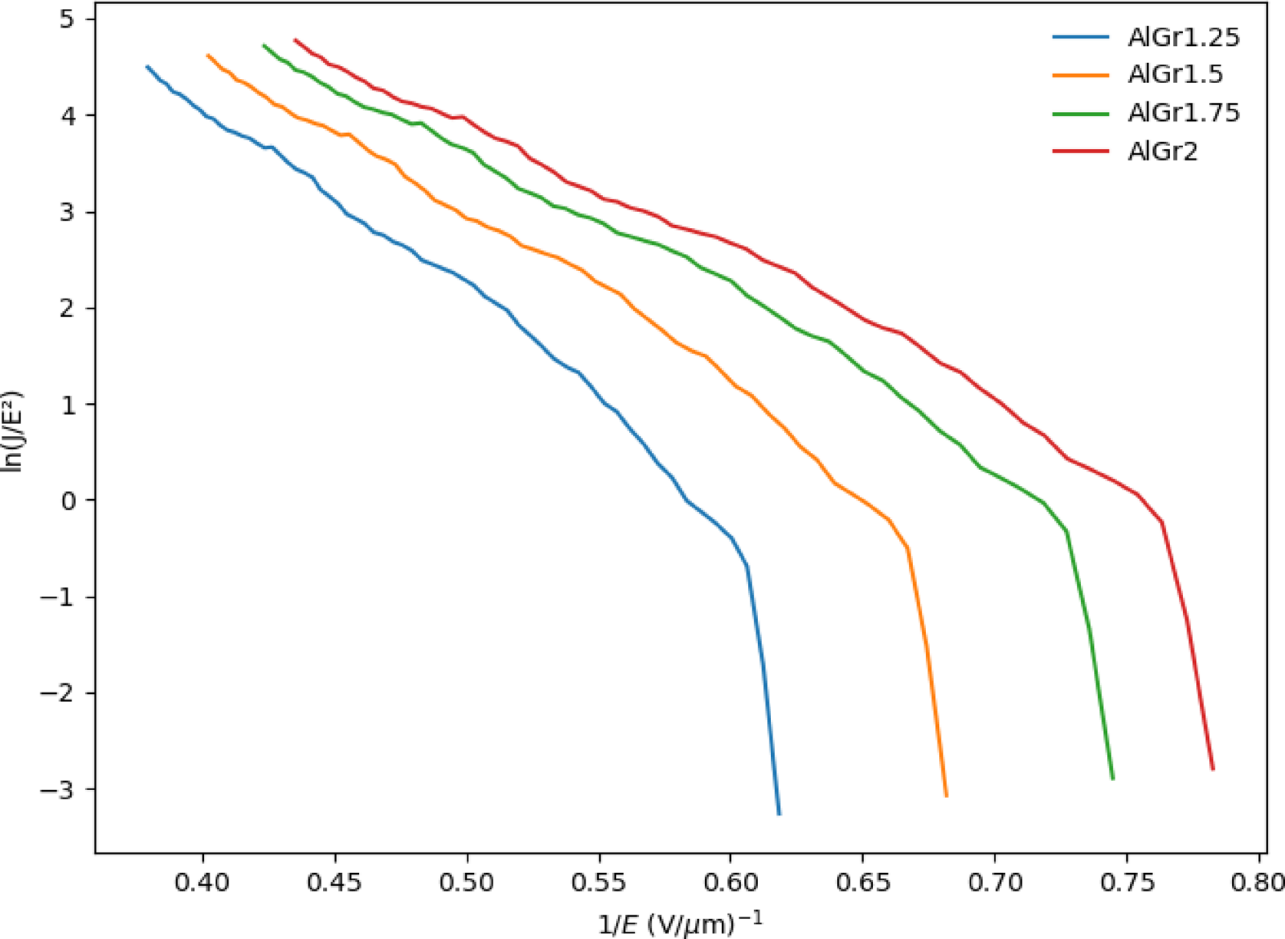
Fowler–Nordheim (F–N) plots of various wt.% of Graphene greater than 1 and less than 2 (1.25%, 1.5%, 1.75%, and 2.0%) inside Aluminium matrix.

Using 10-fold cross-validation in our ensemble model, we predicted the plots shown in Figs. 2, 3 and 4, and Fig. 5 for different graphene weight percentages (1.25%, 1.5%, 1.75%, and 2%) in aluminum. The results of the various predicted features were then analyzed.

Figure 2 illustrates the variation of current density (J), measured in µA/cm², as a function of the electric field (E), measured in V/µm, for the samples AlGr1.25, AlGr1.5, AlGr1.75, and AlGr2. From the Fig. 2, it is evident that as the graphene content in the AlGr composites increases, the field emission properties improve significantly. This enhancement is primarily observed in the reduction of the threshold electric field required for emission. A lower threshold electric field suggests that electrons are emitted more easily, making the material more efficient for field emission applications. Among all the samples, AlGr2, which contains the highest graphene concentration, exhibits the most favorable field emission characteristics. It demonstrates a significantly higher current density at a lower electric field, indicating its superior electron emission capabilities. This improvement can be attributed to the increased number of electron conduction pathways and enhanced electrical conductivity due to the higher graphene content.

Figure 3 further supports these observations by showcasing the stability of the emission current in different AlGr composites. It can be seen that AlGr2 exhibits greater current stability compared to AlGr1.75, AlGr1.5, and AlGr1.25. The reduction in current fluctuations in samples with higher graphene concentrations may be attributed to the presence of stronger and more stable electron-emitting sites, which are less susceptible to degradation caused by ion bombardment. The predicted results suggest that small instabilities in the emission current arise due to the adsorption and desorption of residual gas molecules on the emitter surface. This effect, however, is significantly reduced in samples with a higher graphene concentration, such as AlGr2. The presence of graphene may play a crucial role in minimizing these instabilities by enhancing the robustness of the emission sites and reducing the impact of surface contaminants. Consequently, AlGr2 not only exhibits improved field emission performance but also maintains greater stability over time, making it a promising candidate for advanced electron emission applications.

Figure 4 presents a comparative analysis of the predicted current stability across different graphene weight percentages in the AlGr composite. It can be clearly observed that stability of current increases with increase in graphene concentration.

The Stage 2 results were further refined using 10 K cross-validation, leading to improved predictive performance across models. Among all methods, stacking emerged as the best-performing model giving almost 94% accuracy, achieving an MSE of 105.83, RMSE of 10.28, and the highest R² of 0.93, indicating strong predictive capability and minimal error. CatBoost-GPR followed closely with an MSE of 113.75 and R² of 0.9247, showcasing its reliability in capturing nonlinear dependencies. XGBoost-GPR also exhibited high performance, with an MSE of 118.77 and R² of 0.91918, reinforcing its effectiveness in refining predictions.

Other models, including CatBoost-SVR, LightGBM-GPR, and XGBoost-SVR, demonstrated competitive results, with R² values exceeding 0.90, signifying their robustness in handling complex patterns. However, models such as Bagging-Polynomial and Bagging-GPR had comparatively lower performance, with R² values around 0.82, indicating their limited predictive accuracy in this specific scenario.

The improvements observed in 10 K cross-validation compared to 5 K cross-validation suggest that increasing the validation folds enhanced the model’s generalizability, leading to better stability and reliability in predictions. Stacking, in particular, maintained its superior position, reinforcing its effectiveness in aggregating predictions from multiple base models to minimize variance and bias. Table 5 shows the results of stage 2 with different parameters.

**Table 5:** Shows the different measured matrices of stage 2 with cross validation of 10k.

Model	MSE	RMSE	*R* ^2^	Adj *R*^2^
Stacking	105.8359	10.28766	0.936577	0.934312
CatBoost_GPR	113.7563	10.66566	0.924768	0.922081
XGBoost_GPR	118.7712	10.89822	0.91918	0.916294
CatBoost_SVR	124.0534	11.13792	0.905925	0.902565
LightGBM_GPR	122.3743	11.06229	0.905845	0.902482
XGBoost_SVR	129.603	11.38433	0.895379	0.891643
CatBoost_Polynomial	152.4879	12.3486	0.890368	0.886453
LightGBM_SVR	132.7601	11.52216	0.888728	0.884754
CatBoost_RF	139.3082	11.80289	0.886352	0.882293
XGBoost_RF	145.4651	12.06089	0.885757	0.881676
Voting	134.1475	11.5822	0.880764	0.876506
XGBoost_Polynomial	156.7894	12.52156	0.876171	0.871749
LightGBM_RF	149.0294	12.20776	0.876072	0.871646
Bagging_RF	166.5806	12.90661	0.859916	0.854913
LightGBM_Polynomial	160.6914	12.67641	0.85754	0.852452
Bagging_SVR	148.864	12.20099	0.838697	0.832936
Bagging_GPR	136.8502	11.6983	0.838505	0.832737
Bagging_Polynomial	179.2294	13.38766	0.829093	0.822989

Figure 5. shows the predicted Fowler–Nordheim (F–N) plots of Al, AlGr1.25, AlGr1.5, AlGr1.75 and AlGr2. 

The turn on predicted electric field(E) for aluminium-based metal matrix composites reinforced with graphene (AlGr-MMCs) when J is 10 mA cm^−2^ is shown in Table 6.

**Table 6:** Comparison of the turn-on field between the aluminum–graphene composite and reported composites of aluminum and graphene.

Specimen	Turn On Field Vµm^−1^ at J = 10µAcm^−2^	References
Al nanopowder	4.75 V µm^−1^	[1]
AlGr0.5	2.8 V µm^−1^	[1]
AlGr1.0	2 V µm^−1^	[1]
AlGr1.25	1.81 V µm^−1^	Present Work
AlGr1.5	1.66 V µm^−1^	Present Work
AlGr1.75	1.53 V µm^−1^	Present Work
AlGr2	1.47 V µm^−1^	Present Work

## Field emission mechanism

The field emission performance of aluminium-graphene (AlGr) composites is significantly influenced by the material’s microstructure, electrical conductivity and surface morphology, which are primarily governed by the graphene content. The field emission is a quantum mechanical phenomenon where electrons are emitted from a material’s surface under a strong electric field through tunneling. The applied electric field reduces the surface potential barrier, allowing electrons from the Fermi level to tunnel through the barrier into the vacuum. The emission current density can be described by the Fowler-Nordheim equation, which shows that the emitted current increases exponentially with the applied electric field and inversely with the material’s work function. Achieving low turn-on electric fields and high emission currents requires optimizing the field enhancement factor, which is closely linked to the microstructure and morphology of the composite.

In aluminium-graphene (AlGr) composites, the addition of graphene introduces high electrical conductivity and sharp-edged emission sites that significantly enhance field emission. However, the turn-on electric field shows considerable variation depending on the graphene weight%. Among the studied compositions, AlGr2, containing 2 wt% graphene, exhibits the lowest turn-on electric field, whereas other compositions, including 1.25, 1.5, and 1.75 wt%, show comparatively higher values. This remarkable performance of AlGr2 arises from a delicate balance between conductive network formation, field enhancement and minimal agglomeration. The superior field emission performance of AlGr2 can be attributed to its optimal dispersion and percolation of graphene within the aluminium matrix. At 2 wt% graphene content, the graphene sheets are uniformly distributed, forming a continuous conductive network without significant agglomeration. This interconnected structure reduces resistance and enhances electron mobility, facilitating efficient electron transport to the emission sites. Furthermore, graphene flakes in AlGr 2 are well-aligned to form sharp edges and tips that significantly increase the local electric field, thereby boosting the field enhancement factor and reducing the turn-on field. Additionally, the incorporation of graphene reduces the effective work function of the composite due to graphene’s high electron mobility, which lowers the energy barrier for electron emission.

In contrast, compositions with lower graphene contents, such as 1.25 and 1.5 wt%, exhibit higher turn-on electric fields due to insufficient formation of conductive pathways. The reduced graphene concentration hinders the creation of a continuous network, leading to increased resistance and limited electron transport. Additionally, fewer emission sites with sharp edges result in a lower field enhancement factor, making electron emission more difficult. At slightly higher graphene contents, such as 1.75 wt%, partial agglomeration occurs, creating localized graphene clusters that not only disrupt the conductive network but also induce electric field screening, further increasing the turn-on field. Excessive graphene loading beyond 2 wt% exacerbates this problem as agglomeration becomes more pronounced, causing overlapping of graphene sheets and shielding of emission sites, which reduces the overall field enhancement and compromises emission efficiency. Moreover, under a strong electric field, the potential barrier at the emission site becomes narrow and lowered, allowing electrons from the Fermi level to tunnel through and escape into the vacuum. In AlGr composites, the sharp edges of graphene flakes enhance the local electric field, significantly increasing the probability of tunnelling. This is why compositions with optimally dispersed graphene (like AlGr2) exhibit lower turn-on electric fields and higher emission currents.

Machine learning (ML) techniques were employed to predict the field emission behavior of AlGr composites with different graphene weight percentages. The ML models, including Decision Trees, Support Vector Machines, Neural Networks, Bayesian Models, and Statistical Models, were trained using datasets that encompassed emission current density as a function of the applied electric field and current stability over time. A two-stage machine learning framework was implemented to accurately capture the complex, non-linear relationship between graphene content and field emission characteristics. In the 1 st stage, pure aluminium and AlGr composites with 0.5 wt% and 1.0 wt% graphene was used to train the models. The model evaluation was based on metrics such as R², RMSE, and Adjusted R². The second stage involved refining the top-performing models using advanced techniques, including Gradient-based methods and Ensemble Methods to enhance prediction accuracy. The machine learning models consistently predicted that AlGr2 would exhibit the lowest turn-on electric field, closely aligning with experimental results. This accurate prediction can be attributed to the models’ ability to recognize patterns indicating that 2 wt% graphene offers an optimal balance between conductivity and field enhancement. The models identified that the formation of a well-connected conductive network with sharp emission sites significantly reduces the turn-on field. The sensitivity analysis revealed that field enhancement and conductivity were the most critical factors influencing the emission performance, and both were optimized at 2wt.% graphene content. The advanced ensemble techniques incorporated into the machine learning framework ensured robust predictions by minimizing errors and effectively handling data variability.

The successful prediction of low turn-on electric field values for AlGr2 through machine learning demonstrates the efficacy of data-driven approaches in evaluating and optimizing metal matrix composites for advanced field emission applications. The synergy between experimental analysis and machine learning predictions provides a comprehensive understanding of how graphene content influences emission performance, offering a valuable framework for designing high-performance materials in advanced electronic and energy systems.

The schematic 2 illustrates the field emission mechanism of aluminium-graphene (AlGr) composites with varying graphene weight percentages (1.25%, 1.5%, 1.75%, and 2.0 wt%) under an applied electric field. The setup consists of a cathode positioned above the anode, with the anode comprising AlGr composites arranged in a gradient of graphene content. The electric field lines are depicted as parallel lines between the cathode and the anode, representing the uniformity of the applied electric field.

**Schematic 2:**
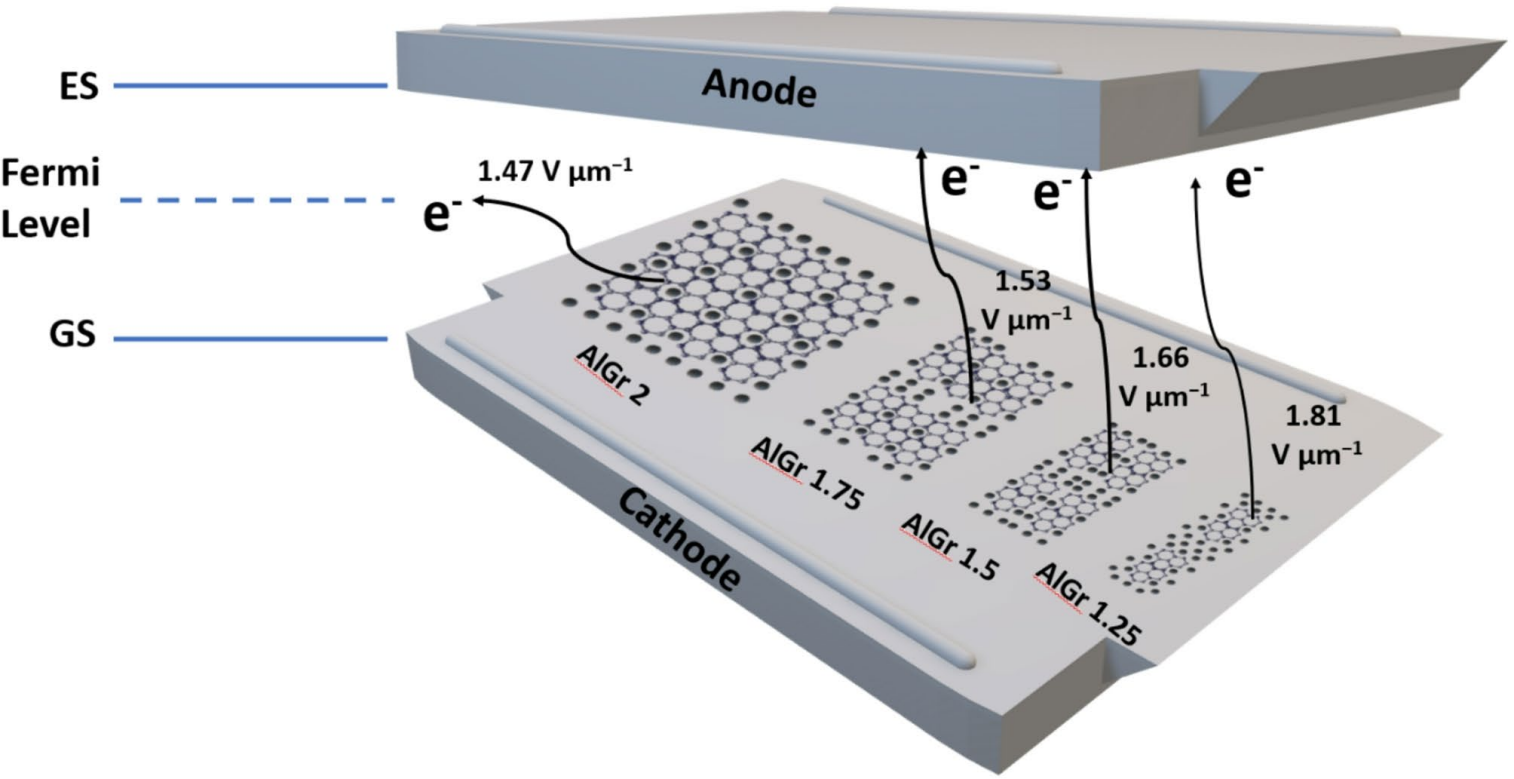
Field emission mechanism for AlGr 2, AlGr 1.75, AlGr 1.5 and AlGr 1.25.

In the field emission mechanism of AlGr composites, the Fermi level plays a crucial role in determining the emission efficiency. The Fermi level represents the highest energy level occupied by electrons at absolute zero temperature. In the case of AlGr 2, the well-dispersed graphene network and strong interfacial bonding between aluminium and graphene result in efficient electron transfer and uniform distribution of the electric field at the emission sites. This optimal configuration ensures that electrons near the Fermi level are most likely to tunnel through the potential barrier, as the localized electric field enhancement at graphene edges reduces the barrier width and height. Consequently, electrons from the Fermi level have sufficient energy to undergo quantum tunnelling without needing to occupy higher energy levels. In contrast, AlGr1.75, AlGr1.5, and AlGr1.25 composites exhibit less uniform graphene dispersion, leading to agglomeration or clustering. This results in localized field screening and uneven electric field distribution, causing the electrons to acquire additional kinetic energy to overcome irregular barrier heights. Consequently, electrons tend to occupy higher energy states above the Fermi level, as the uneven electric field necessitates higher excitation energies for emission. This results in a higher turn-on electric field and reduced emission efficiency. The difference in Fermi level involvement between AlGr2 and other composites underscores the importance of achieving a uniform graphene distribution to minimize energy losses and optimize field emission performance.

## Limitations of the study

Despite the promising results and the novel application of machine learning for predicting field emission behavior in graphene-reinforced aluminium composites, this study has a few important limitations that should be acknowledged. The most significant limitation lies in the reliance on extrapolated predictions for compositions containing more than 1 wt% graphene. The models were trained exclusively on experimentally obtained data for pure aluminium, AlGr0.5, and AlGr1.0, and while high predictive performance was achieved for higher concentrations (AlGr1.25–AlGr2.0), these predictions remain unverified in the absence of corresponding experimental validation. Consequently, while the models indicate robust trends and offer valuable guidance for future material design, the outputs beyond the 1 wt% experimental limit must be treated as informed estimates rather than definitive conclusions. Another limitation concerns the scale and scope of the dataset. With only 65 datapoints per composition in the JE dataset and 1800 in the Stability dataset, the sample size is relatively modest for data-driven modeling. While ensemble and probabilistic models such as GPR, SVR, and Stacking effectively captured nonlinear patterns and generalized well under cross-validation, the training data may still restrict model complexity, particularly for deep learning approaches. Moreover, these models do not incorporate microstructural descriptors such as defect density, grain size, or interfacial morphology, which are known to influence field emission but are currently unavailable in the dataset. Additionally, the study focuses exclusively on Al–graphene composites. As a result, the trained models are not immediately transferable to other metal matrix or reinforcement systems without retraining and appropriate domain adaptation. The current framework is therefore material-specific, and its predictive power may not generalize to dissimilar systems such as Cu–CNT or Mg–SiC composites. Furthermore, although both 5-fold and 10-fold cross-validation strategies were employed to ensure robustness, the inherent variability in experimental conditions and environmental noise cannot be fully captured by synthetic data partitions alone.

These limitations, while not detracting from the core findings, highlight the need for continued work involving larger, more diverse datasets, experimental validation at higher graphene loadings, and the integration of additional physical parameters. Addressing these areas would significantly strengthen the generalizability, interpretability, and practical impact of machine learning-based approaches in material performance prediction.

## Conclusion

In this paper, we developed a two-stage machine learning framework to predict the field emission performance of graphene-reinforced aluminum-based metal matrix composites (AlGr- MMCs). Given the limitations of experimental methods for graphene concentrations above 1 wt%, we leveraged machine learning to identify complex patterns and establish nonlinear relationships. Stage 1 involved evaluating multiple models, with Gaussian Process Regressor (GPR), Support Vector Regression (SVR), Random Forest, and Polynomial Regression emerging as top performers. In Stage 2, we refined predictions using gradient-based methods (XGBoost, LightGBM, CatBoost) and ensemble techniques (Stacking, Voting, Bagging). Stacking consistently achieved the best results, demonstrating the lowest error and highest accuracy across both 5 K and 10 K cross-validations. This study provides a reliable computational framework for optimizing material properties in high-performance electronics and energy applications. Among the studied compositions, AlGr 2, containing 2 wt% graphene, exhibits the lowest turn-on electric field, whereas other compositions, including 1.25, 1.5, and 1.75 wt%, show comparatively higher values. This remarkable performance of AlGr2 arises from a delicate balance between conductive network formation, field enhancement and minimal agglomeration. The superior field emission performance of AlGr2 can be attributed to its optimal dispersion and percolation of graphene within the aluminium matrix. Future work could expand datasets and explore deep learning approaches to further enhance predictive accuracy.

## Data Availability

All raw experimental data used in this study were obtained from the authors’ prior work (Pradhan, S. K., Pandiyarajan, K., Patil, S., Chavan, P. G., Lobato, R. L. M., Ribeiro-Soares, J. & Late, D. J. Field emission performance of graphene-incorporated aluminum-based metal matrix composite. Nanoscale Adv. 7,614-620 (2024)), where the corresponding author of this paper was also the corresponding author. The raw data to construct the processed ML training datasets, can be made available upon reasonable request from the corresponding author.
